# Understanding Nasal Polyposis: The Roles of Ion Channels, Inflammation, Ionocytes, and Prostaglandin E2—The I^3^PGE_2_ Hypothesis

**DOI:** 10.3390/jcm15176908

**Published:** 2026-09-07

**Authors:** César Picado, Jordi Roca-Ferrer

**Affiliations:** 1 Faculty of Medicine and Health Sciences, University of Barcelona, 08036 Barcelona, Spain; 2 Institut d’Investigacions Biomèdiques August Pi I Sunyer (IDIBAPS), 08036 Barcelona, Spain; jrocaf@recerca.clinic.cat; 3 Centro de Investigaciones en Red de Enfermedades Respiratorias (CIBERES), 28029 Madrid, Spain

**Keywords:** chronic rhinosinusitis, cystic fibrosis, inflammation, ion channel, ionocyte, nasal polyp

## Abstract

Nasal polyposis is a multifactorial disorder arising from complex interactions among cellular, molecular, and inflammatory mechanisms. The Cystic Fibrosis Transmembrane Conductance Regulator (CFTR) and other ion channels are essential for epithelial ion transport, mucosal hydration, and barrier integrity in the nasal airway. Prostaglandin E_2_ (PGE_2_) acts not only as an inflammatory mediator but also as a regulator of ion exchange through CFTR-dependent and CFTR-independent pathways. Ionocytes, specialized epithelial cells that regulate ion balance and fluid secretion in the respiratory tract, are closely associated with CFTR function. Both eosinophilic and non-eosinophilic inflammation may alter ionocyte abundance and function, thereby reducing normal CFTR activity. Chronic rhinosinusitis with nasal polyps (CRSwNP) is also characterized by diminished PGE_2_ production. The I^3^PGE_2_ hypothesis proposes that CRSwNP results from the combined effects of ion channel dysfunction, persistent inflammation, altered ionocyte number and function, and impaired PGE_2_ synthesis. Together, these abnormalities disrupt nasal physiology and promote polyp formation. We hypothesize that corticosteroids and biologic therapies may improve CRSwNP by reducing inflammation, restoring ion channel activity, improving ionocyte function, and recovering PGE_2_ production. These effects enhance hydration and mucociliary clearance, limit mucus accumulation, restore epithelial homeostasis and mucosal host defense, and ultimately contribute to the reduction or resolution of nasal polyps.

## 1. Introduction

Nasal polyps (NPs) are inflammatory growths in the nasal mucosa, typically found bilaterally in the anterior ethmoid region and middle meatus. They are often linked to chronic rhinosinusitis (CRS), which is divided into CRS with NPs (CRSwNP) and CRS without NPs (CRSsNP). CRSwNP patients generally show more severe sinus disease on CT scans compared to CRSsNP patients [1].

NPs are usually diagnosed with nasal endoscopy, which directly reveals polypoid lesions [1]. However, some patients with CRSsNP who have surgery are found to have polyps in their sinuses not previously detected by endoscopy [2].

CRSwNP subtypes include aspirin-exacerbated respiratory disease (AERD), also known as non-steroidal exacerbated respiratory disease (N-ERD), CRSwNP associated with cystic fibrosis (CF) [1], and central compartment atopic disease (CCAD) [3]. CCAD affects the middle and superior turbinate turbinates, and posterior superior nasal septum, has strong ties to inhalant allergens [3].

A meta-analysis of observational studies found CRS prevalence at 8.71% globally and CRSwNP at 0.65%. Rates were higher in Europe, adults, smokers, people with obesity, and those with asthma or nasal septal deviation compared to other groups [4].

CRS has been reported in nearly all patients with CF, while NP are diagnosed with a variable proportion of patients, ranging from 32% to 44% [5].

Tissue inflammation in CRSwNP and CRSsNP involves distinct inflammatory endotypes, classified by main immune cells and cytokines. CRSwNP typically features eosinophilic inflammation, while CRSsNP is usually neutrophilic or mixed [6].

The prevalence of these endotypes varies significantly, influenced by assessment methods and showing distinct regional differences—especially between Western and East Asian countries—which are shaped by genetic, environmental, and lifestyle factors [6].

Specific cytokines define CRS endotypes: T1 is marked by IFN-γ, T2 by IL-4, IL-5, IL-13, and eosinophils, and T3 by IL-17 and IL-22. Some patients show mixed T1/T2/T3 patterns with overlapping clinical features [6,7]. CRSwNP is mainly T2-driven, whereas CRSsNP usually shows combined T1, T2, and T3 cytokine expression [6,7].

Innate lymphoid cells (ILCs), comprising three main types (ILC1, ILC2, ILC3), contribute to CRS by releasing distinct cytokines corresponding to T1, T2, and T3 inflammatory endotypes [6,7].

Adaptive immunity is also disrupted in CRS, especially CRSwNP. CD4+ T cells from European patients mainly produce type 2 cytokines, whereas those from Asian patients tend to release type 1 and type 17 cytokines. Treg cell alterations are reported, but findings on their abundance in CRSwNP remain inconsistent [1,6,7].

Studies also reported increased numbers of lymphocyte B cells, basophils and mast cells in CRSwNP. Accumulation of B cells is associated with elevated local production of immunoglobulins (IgA, IgG, and IgE) [1,6].

Viruses and bacteria are linked to CRS, with or without NPs. CRS patients often have higher levels of rhinovirus, parainfluenza, influenza, and RSV in nasal secretions than controls. *Staphylococcus aureus* (SA) and *Pseudomonas aeruginosa* (PA) are common bacterial colonizers; patients with SA frequently develop specific IgE antibodies [1,8].

Nasal microbiomes differ widely among individuals and countries, but both CRS patients and healthy people share core bacteria: *Corynebacterium*, *Staphylococcus*, *Streptococcus*, *Haemophilus*, and *Moraxella*. In CRS, *Corynebacterium* is notably less abundant, while *Streptococcus* shows a minor, non-significant increase, and other core bacteria levels remain stable [8].

Lower levels of epithelial antimicrobial proteins like betadefensins and lysozyme, may allow more pathogens and alter the normal microbiome in CRS patients [1,6].

Upon exposure to different insults, nasal epithelial cells in CRS secrete epithelial-derived cytokines—interleukin-25 (IL-25), interleukin-33 (IL-33), and thymic stromal lymphopoietin (TSLP)—collectively referred to as alarmins. These mediators subsequently activate type 2 innate lymphoid cells (ILC2s) as well as diverse other immune cell populations, thereby promoting the inflammatory cascade associated with CRS [9].

In CCAD airway mucosa, inflammation is marked by local IgE presence, a slight rise in eosinophils, and lower levels of cytokines like IL-5, IL-13, IL-6, and IFN-γ when compared to other forms of CRSwNP or N-ERD [10].

CF polyps typically show enlarged glandular ducts, abundant mucous glands, more plasma cells and mast cells, and fewer eosinophils than non-CF polyps. However, inflammatory patterns vary, with some cases dominated by neutrophils or mixed neutrophilic–eosinophilic infiltrates [11,12].

Overall, these findings indicate that CRS, regardless of the presence of NPs, develops because of a multifaceted and intricate series of events. This complexity gives rise to different patterns of inflammation, each characterized by distinct immunological and cellular mechanisms.

## 2. Hypotheses Explaining Crswnp

Several hypotheses have been put forward and largely discussed to explain the origins of CRSwNP, exploring the play role by genetic predisposition, allergy, altered innate and adaptive immune responses, microbiome and infections, vitamin D deficiency, hypoxic environment, fungus allergy, increased activation of the coagulation cascade, physical events (Bernoulli’s phenomenon), embryonic olfactory tissue remnants, and epithelial dysfunction within the airway mucosa [13,14,15,16,17]. We summarized some of the most frequently highlighted.

*Allergy hypothesis*. Allergies were once thought to be the main cause of NPs due to persistent eosinophilic inflammation, associated with locally produced IgE from airborne substances, especially in those with atopic disease. However, studies show NPs are often linked to non-atopic conditions, suggesting other factors are more influential. A systematic review of CRSwNP found that seven out of 18 studies showed no relationship between allergy and CRSwNP; 10 studies demonstrated a relationship between allergy and CRSwNP; and one study was equivocal [18]. The recent description of CCAD has renewed interest in allergic mechanisms and their role in NPs development [3].

*Chronic Eosinophilic Inflammatory Response*. This theory states that ongoing eosinophilic inflammation, typically marked by local eosinophilic and detection of IL-5 in polyp’s tissue independent of any allergic background results in chronic hyperplastic sinusitis eventually evolving to NP. However, neutrophilic and mixed patterns are also associated with inflammatory phenotypes other than the characterized by predominant isolated eosinophil nasal mucosa infiltration [16].

*Staphylococcus aureus* (SA) *and the superantigen hypothesis*. Persistent colonization of the airway epithelium by SA is associated with CRS. When SA remains within the nasal cavity, it can induce substantial inflammation, primarily through exotoxins functioning as superantigens. These superantigens provoke widespread activation of T lymphocytes, resulting in a significant release of type 2 cytokines. This increase in cytokine activity activates B cells and generates a local polyclonal IgE response. Ultimately, this process leads to pronounced eosinophilic inflammation in the nasal mucosa, which frequently precedes the formation of nasal polyps. However, there is insufficient evidence to conclude that these sequential events alone account for the origin of NPs or whether they serve merely as contributory incidental factors [16].

*Inflammatory-bioelectric theory*. As proposed by Bernstein and coworkers [19,20], it provides an explanation for the development of NPs based on disruptions in ion transport within the nasal mucosa. According to this theory, mucosal injury results from turbulent airflow and recurrent infections, which cause repeated cycles of inflammation and ulceration in the nasal lining. These injuries impair the secretion of chloride ions while increasing the reabsorption of sodium ions, leading to an imbalance in electrolyte transport. This imbalance draws excess water into the mucosal cells, resulting in tissue edema and, ultimately, the formation of nasal polyps. The process is further amplified by inflammatory mediators that exacerbate these changes in ion transport. Bernstein and coworkers [19,20], suggest that the underlying disorder in electrolyte movement may stem from impaired function of the Cystic Fibrosis Transmembrane Conductance Regulator (CFTR) protein in the nasal epithelium. CFTR is an anion channel regulated by cAMP. Supporting this hypothesis, studies have shown that epithelial cells from NPs display significantly higher transepithelial potential differences compared to healthy tissue, which is consistent with altered ion transport mechanisms in polyp formation. Since this theory emphasizes inflammation as the trigger for electrical dysfunction, corticosteroids are the first-line treatment to reduce swelling and restore barrier integrity [19,20].

*Epithelial Damage Theory.* The epithelial damage theory is a widely accepted explanation for the development of chronic inflammatory diseases across various organ systems, including the skin, upper and lower airways, and the gastrointestinal tract [21,22,23,24], According to this hypothesis, injury to the epithelial tissue—such as the nasal mucosa—can occur due to bacterial or viral infections or ongoing exposure to environmental irritants. In the context of CRSwNP, this epithelial damage disrupts the normal barrier function of the nasal lining. As a result, a cycle of chronic inflammation is initiated and perpetuated by alterations in the local airway microbiome. The continued presence of inflammation interferes with the typical repair mechanisms of the tissue, ultimately leading to incomplete healing. Instead of restoring the normal mucosal architecture, this failed repair process results in submucosal prolapse, contributing to the formation of nasal polyps [23].

Although several hypotheses exist about CRSwNP mechanisms, most lack solid scientific evidence [13,14,15,16,17]. Except for the inflammatory bioelectric theory, research has rarely addressed the link between CFTR abnormal function and CRSwNP pathogenesis [19,20]. However, the inflammatory bioelectric theory does not provide any mechanism to explain the abnormal CFTR functioning apparently associated with chronic nasal inflammation.

This manuscript explores how dysregulated airway hydration may contribute to CRSwNP in both CF and non-CF individuals. We propose that impaired hydration drives chronic inflammation, infection, and nasal polyp formation through a shared pathogenic pathway, offering a framework for identifying mechanisms common to CF-related and non-CF CRSwNP.

## 3. Ion Channels, Ionocytes, Inflammation, and Prostaglandin E_2_: The I^3^PGE_2_ Hypothesis

### 3.1. The CFTR and Other Ion Channels 

The CFTR protein plays a crucial role in regulating salt and water homeostasis across epithelial surfaces, helping mucus secretions stay well-hydrated and less thick. This osmotic balance is maintained by several coordinated actions: CFTR moves negatively charged chloride (Cl^−^) and bicarbonate (HCO3^−^) ions out of the cell and into the airway lumen. To keep electrical neutrality, positively charged sodium (Na^+^) ions follow through the paracellular pathway, between cells. As salt accumulates outside the cell, an osmotic gradient forms, drawing water out—either through the cell membrane via aquaporins or between cells—to moisten the surface. Additionally, CFTR serves as a “brake” on the Epithelial Sodium Channel (ENaC), inhibiting ENaC to prevent excessive reabsorption of water by the cell [25]. CFTR also regulates other Cl^−^ channels and transporters, some of which belong to the SLC26 family [25].

CF is caused by mutations in the *CFTR* gene, with over 2500 different variants identified to date. These mutations are primarily categorized into five or six functional classes based on how they disrupt the production or function of the CFTR protein [26].

When CFTR is absent or faulty, the regulation of salt and water transport across the epithelial surface is disrupted. Chloride ions remain trapped inside the cell, preventing Na^+^ and water from moving to the airway surface. The loss of CFTR’s inhibitory effect on the ENaC causes ENaC to become overactive, further increasing the reabsorption of salt and water into the cell, which contributes to dehydrate airway surfaces and produces thick mucus. Regulation involves protein interactions, chloride concentration changes, and ENaC trafficking [27,28]. Nevertheless, the mechanisms involved in the relationship between CFTR and ENaC are still unclear due to contradictory results in different studies [27].

This sequence of events resulting from CFTR mutations leading to altered airway surface liquid (ASL) causes the mucus layer to collapse onto the underlying cilia, impairing their ability to move and clear mucus from the airway [25].

Following a period of sterile inflammation [29], SA and PA infections and bacterial dysbiosis maintain airway inflammation, abnormal mucus, and lung injury—main causes of cystic fibrosis complications and deaths [30]. These factors lead to nearly universal CRS often associated with NP in CF [5].

Mutations in *CFTR* that do not fully fit the classic diagnostic criteria for CF are linked to various conditions known as CFTR-Related Disorders (CFTR-RD), such as Congenital Bilateral Absence of the Vas Deferens (CBAVD) and chronic pancreatitis. Studies reported that both CRS and NPs are found more frequently in patients with CFTR-RDs than in the population [30,31].

Numerous studies have shown that individuals possessing a single CFTR mutation—commonly termed carriers—exhibit a heightened risk of developing sinonasal disease relative to the general population. CFTR variants cited in the literature include ΔF508 (F508del), R117H, 3272-26A>G, and the 5T allele (IVS8-5t) [32,33,34,35].

Recent advances have transformed CF treatment, with therapies targeting specific CFTR protein defects. These include correctors, which help the CFTR protein fold correctly; potentiators, which keep the CFTR channel open for chloride ions; and amplifiers, which boost overall CFTR protein production [36]. Modern treatments often used multiple modulators such as the combination of elexacaftor/tezacaftor/ivacaftor and of vanzacaftor/tezacaftor/deutivacaftor to address various defects at once [36]. Triple therapy results in a significant improvement in clinical symptoms, reduction in exacerbations, improved lung function, and decreased systemic inflammation [37,38,39,40].

**Figure 1:**
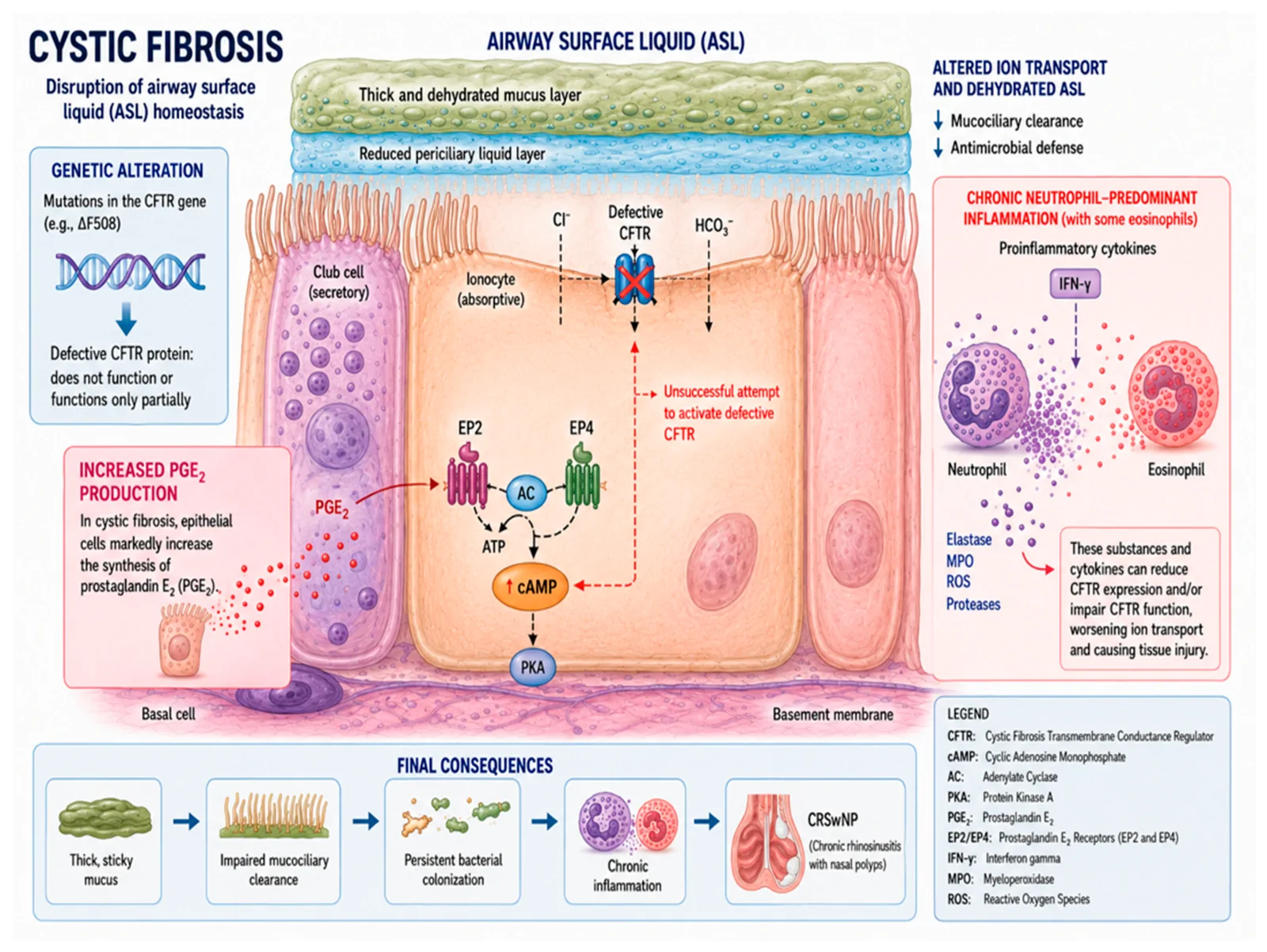
Cystic fibrosis (CF) is caused by mutations in the *CFTR* gene. CFTR protein maintains salt and water balance across epithelial surfaces, hydrating mucus by secreting chloride into the airway lumen; sodium and water follow osmotically. CFTR also inhibits the epithelial sodium channel (ENaC), limiting water reabsorption. When CFTR is absent or defective, chloride, sodium, and water fail to reach the airway surface, while ENaC becomes overactive and increases salt and water reabsorption into cells. Inflammation may further reduce CFTR activity. This dehydrates the airway surface and produces thick mucus. CFTR regulates PGE_2_, and PGE_2_ strongly activates CFTR. In CF, PGE_2_ is markedly upregulated in an unsuccessful attempt to activate impaired CFTR. CFTR modulator therapies rapidly improve sinonasal symptoms and radiological sinus opacification and normalize PGE_2_ production.

Highly effective triple CFTR modulator therapies, such as the combination of elexacaftor/tezacaftor/ivacaftor and vanzacaftor/tezacaftor/deutivacaftor, have significantly improved the management of CRS and NP in CF. Patients typically report a “dramatic improvement” in sinonasal symptoms within 7 to 28 days of starting therapy. CT scans have shown a 90% improvement in sinus opacification [41,42,43,44,45,46]. However, upon discontinuation of therapy, NPs tend to recur rapidly [47] (Figure 1).

Besides CFTR and ENaC other ion channels involved in ASL regulation are: CaCC (Ca-activated chloride channels): notably TMEM16A, which contributes to apical chloride secretion, and CLC2 (Chloride channel 2) and CNG (Cyclic nucleotide-gated channels), apical channels that support ENaC function [48].

ENaC is made up of three related subunits: α, β, and γ. These subunits are expressed at higher levels in NPs than in the nasal mucosa [49]. However, research results vary on which ENaC subunit is most common in NPs—one study points to α [49], another to β [50], and a third highlights γ [51]. There is also a link between Na+ absorption and γ ENaC mRNA expression in NPs [39].

ENaC is modulated by proteases such as neutrophil elastase, a process often intensified during inflammation [52]. The secreted protein short palate lung and nasal epithelial clone 1 (SPLUNC1) expression is inhibited by Th2 cytokines, such as IL-4 and IL-13. Polyp tissues exhibited significantly decreased expression of SPLUNC1. Moreover, the eosinophilic CRSwNP subset exhibited significantly decreased SPLUNC1 expression as well as significantly increased IL-4 and IL-13 mRNA levels, compared with the non-eosinophilic subset. A vicious cycle occurs where the inflammatory milieu reduces SPLUNC1, and the loss of SPLUNC1 allows for further uncontrolled inflammation. As SPLUNC1 normally acts as an inhibitor of ENaC, a decrease in SPLUNC1 causes overactivation of ENaC [52,53].

Taken together, these findings suggest that increased ENaC expression and activation may enhance transepithelial sodium and water reabsorption, thereby depleting the airway surface liquid. This process may contribute to epithelial dehydration and to the tissue edema commonly observed in NPs.

TMEM16A (also known as Anoctamin-1) is a calcium-activated chloride channel that acts as a critical regulator of mucus hypersecretion and epithelial hydration. Its expression is upregulated by IL-13 and IL-4, which are hallmarks of the inflammatory environment in nasal polyps. While some studies show increased expression, research also indicates that patients with CRSwNP may have impaired TMEM16A-mediated chloride secretion. This dysfunction may lead to reduced airway surface hydration and impaired mucociliary clearance [54,55].

The Na^+^/K^+^/2Cl^−^ cotransporter 1 (NKCC1) plays a critical role in the development of mucosal edema. It acts as a primary chloride importer that, when overexpressed, drives the accumulation of ions and water within the tissue. In NPs, it is found at significantly higher levels throughout the epithelial layer compared to healthy nasal tissue. By increasing the concentration of intracellular chloride above its equilibrium, NKCC1 creates an osmotic gradient that draws water into the cells and surrounding interstitial tissue. This process leads to tissue swelling characteristic of polyp growth [56].

Notably, intranasal frusemide inhibits NKCC1 and reverses the osmotic gradient in nasal tissue, and thereby effectively reduces edema, relieves obstruction, and prevents postsurgical recurrence as effectively as corticosteroids [57,58,59,60].

Collectively, these findings indicate that impaired functioning of several major ion channels contributes to deficiencies in ASL homeostasis in NPs. Ion channels such as CFTR, ENaC, TMEM16A, and NKCC1 are critical for regulating anion and fluid transport across the airway epithelium. Disruption of these channels may alter epithelial surface properties and compromise airway hydration. Although further studies are needed, these abnormalities provide a plausible mechanism linking ion transport dysfunction to the pathophysiological features involved in NP development (Table 1).

### 3.2. Cells (Secretory and Ionocytes)

Single-cell RNA sequencing and lineage tracing identify common airway epithelial cells—basal, club, ciliated, goblet—as well as rare types like pulmonary neuroendocrine cells, tuft cells, and ionocytes. Distinct secretory cells include goblet cells (secreting mucin), serous cells (providing water, enzymes, and antimicrobial substances), and club cells (producing macromolecules and acting as progenitors for airway repair) [61,62,63].

Ionocytes, originally referred to as “chloride cells,” are specialized mitochondria-rich cells that play a pivotal role in regulating ions and water balance within an organism. While these cells have long been recognized for their osmoregulatory functions in aquatic animals, their presence in the human lung was only recently discovered [62,63].

Ionocytes have a distinct tadpole appearance under a microscope, with a wider head and a narrowing tail. The head contains an oval nucleus and is typically situated at the base of the epithelium, while the thin tail extends upward to the surface. They often gather in groups between ciliated cells, especially in areas with high CFTR expression [64,65]. Ionocytes can be identified by the expression of specific genes, including FOXI1 and ASCL3 [61,62,63].

Club cells are the dominant source of functional CFTR expression in both large and small airways. However, CFTR expression levels in these cells are lower than in ionocytes [65]. Pulmonary ionocytes comprise approximately 1% of the total cell population in the airways. Research has demonstrated that CFTR transcripts within ionocytes represent nearly 50% of all CFTR transcripts found in the airway epithelium [61,62,63]. This finding underscores the importance of ionocytes in terms of CFTR abundance at the transcript level. However, other studies indicate that the larger population of secretory cells collectively provides a greater amount of CFTR, even though individual ionocytes have high transcript levels [66].

The way club cells and ionocytes control ion exchange remains a topic of active discussion [65,66,67,68,69,70,71]. Although club cells are thought to be the main contributors to secrete airway fluid [65], new research has highlighted that ionocytes, despite their lower abundance, possess distinct mechanisms and functions in ion regulation that set them apart from club cells [66,67,68,69,70,71]. Beyond maintaining salt balance, ionocytes are also instrumental in controlling the acidity of surface liquids in the lungs and other tissues [68].

This evolving understanding emphasizes that club cells and ionocytes act through separate pathways to manage ion exchange, and their contributions may differ in both healthy and diseased airway environments [40,69,70,71].

The distribution and morphology of club cells and inocytes in CF and non-CF NPs have been examined in a few studies.

Specific counts of club cells and ionocytes in the nose of CF patients are limited. Nasal swabs were collected before and three months after triple therapy in children with CF and compared with healthy controls. Ionocytes showed the highest CFTR expression, followed by secretory, basal, and ciliated cells. Children with CF had fewer CFTR-expressing cells at baseline, particularly among secretory cells, but this increased to normal levels after treatment. Ionocyte CFTR cell proportions did not decrease in CF patients [40]. The reasons for these differences in CFTR expression across cell types remain unclear. In another study the main percentage of ionocytes in nasal brushings was 3% in both CF subjects and healthy controls [72]. This abundance was higher than the 1% reported in the lower airways of healthy subjects [61,62]. As expected, the number of ionocytes in most CF samples were devoid of CFTR staining. Although much lower than in healthy controls, CFTR signal in CF patient with mild mutations was found significantly higher than in those patients with severe mutations [72].

A study of non-CF CRSwNP classified by neutrophil (N) and eosinophil (E) infiltration into four endotypes (N^−^E^−^, N^+^E^−^, N^−^E^+^, and N^+^E^+^) showed that club and ciliated cells were significantly reduced across all endotypes, whereas basal and goblet cells increased in neutrophilic and eosinophilic CRSwNP, respectively. Protein expression of FOXJ1, an ionocyte marker, did not differ significantly between CRSwNP and controls. Because club cells are essential for airway repair and maintenance, their reduction in NPs may compromise mucosal health and function [73]. Consistent with this finding, nasal secretions from patients with CRSwNP showed significantly lower mean concentrations of Clara cell protein 16 (CC16) than controls [74].

However, other studies found that the spatial distribution of ionocytes shifts notably in the context of CRS and NPs. NPs are characterized by a greater proportion of ionocytes that either lose their CFTR expression or no longer exhibit their typical tadpole morphology. These results likely explain the reduced levels of CFTR protein and mRNA found in polyp tissue relative to healthy nasal mucosa. In healthy nasal mucosa, CFTR is predominantly localized at the apical region of the cell membrane, supporting ion transport. Conversely, in NPs, the protein frequently relocates to a cytoplasmic or perinuclear position, resulting in its intracellular sequestration and impaired function [64].

In summary, proper ion transport and ASL balance depend on the coordinated activity of club cells and ionocytes. Reduced club cell numbers, together with altered CFTR expression in ionocytes, may impair epithelial ion transport and contribute to the pathophysiological mechanisms underlying CRSwNP (Table 1). However, current evidence directly linking ionocyte dysfunction to CRSwNP remains limited. Further studies are therefore needed to clarify both the extent of ionocyte involvement and its biological significance in disease development.

### 3.3. Inflammation 

Research has examined the impact of inflammation on the airway distribution of club cells and ionocytes, as well as their regulation and physiological function in maintaining homeostasis of ASL [75,76].

A recent study showed that airway epithelial cells from patients with non-eosinophilic asthma, including paucigranulocytic and neutrophilic subtypes, have markedly reduced CFTR function and fewer CFTR-expressing ionocytes. No ionocytes were detected in neutrophilic asthma, and only very few were found in paucigranulocytic asthma, whereas ionocytes accounted for approximately 2% of cells in both eosinophilic asthma and healthy controls. Analysis of CFTR distribution across airway epithelial cell types showed that, in healthy donors, ionocytes were the main CFTR-expressing cells, although CFTR transcripts were also present in club cells and in the secretory/goblet lineage. In neutrophilic asthma, ionocyte expression was lost, while residual CFTR transcripts persisted in club cells and in the secretory/goblet lineage; these non-ionocyte CFTR transcript levels remained relatively stable compared with healthy donors. The reduction in ionocytes and CFTR expression appears to be driven by inflammatory cytokines associated with non-eosinophilic profiles, particularly IFN-γ [75]. This acquired CFTR dysfunction in non-eosinophilic asthma may impair ASL hydration and contribute to mucus obstruction [75].

In contrast to results in non-eosinophilic asthma [75], IL-13 (T2 cytokine)-stimulated airway epithelia displayed an overall increase in CFTR expression due to an increase in CFTR-expressing secretory cells and a concordant increase in CFTR-mediated chloride current, despite the loss of CFTR-expressing ionocytes. IL-13-treated cells resulted in less apical liquid absorption and therefore an increase in ASL volume, a finding that is consistent with the combined effects of ionocyte absorbing fluid and secretory cells secreting fluid working together to regulate ASL volume. Importantly, the defect in liquid absorption observed with the loss of CFTR-expressing ionocytes can be reversed. Restoration of ionocyte numbers—achieved by virally overexpressing the ionocyte-specific transcription factor FOXI1—successfully rescues the ability of the epithelium to absorb apical liquid, normalizing ASL volume [76].

To further investigate ionocyte CFTR expression, lung samples from asthma patients and control subjects were obtained through two ways. In one study samples were obtained from a deceased donor without lung disease and from an asthma patient who died from an asthma exacerbation. In the control donor a strong CFRT labeling at the apical surface of ionocytes was observed. In contrast, apical CFTR labeling was not detected in most ionocytes in asthma samples.

The second study used bronchial biopsies of lower airway biopsies from controls or asthma patients. Ionocytes numbers were similar in both groups. However, the overall percentage of inocytes with CFTR was reduced in asthmatic samples, with 2 different populations: one population expressed CFTR at levels comparable to controls, while the other population of ionocytes lacked CFTR labelling. When compared CFTR-positive numbers with spirometry data it was found that the CFTR-positive ionocyte numbers correlate with worsening airflow obstruction. Collectively these observations suggest that in IL-13 driven diseases such as asthma, loss of CFTR expression in ionocyte may contribute to the abnormal regulation of ASL homeostasis and thereby to airway obstruction [76].

Although studies differ on the precise roles of CFTR-expressing club cells and ionocytes, current evidence suggests that inflammation disrupts ASL regulation. We propose that this dysregulation may contribute substantially to the pathogenesis and progression of airway diseases such as asthma and CRSwNP, which often coexist.

### 3.4. Prostaglandin E2 (PGE_2_)

In the human body PGE_2_ is produced from arachidonic acid (AA) via a multi-step enzymatic sequence. The enzyme phospholipase A2 releases AA from cell membranes, which is converted to prostaglandin G_2_ (PGG_2_) and then into prostaglandin H_2_ (PGH_2_) by cyclooxygenase-1 (COX-1) (constitutive) or COX-2 (inducible during inflammation) enzymes. Finally, PGH_2_ becomes PGE_2_ through specific prostaglandin E synthases like mPGES-1, mPGES-2, or cPGES [77].

PGE_2_ acts through four G protein-coupled receptors (GPCRs) called EP receptors (EP1–EP4), each encoded by separate genes and linked to specific signaling pathways. In the lungs, EP2 and EP4 mediate anti-inflammatory and bronchodilator effects, relaxing airway smooth muscle, inhibiting mast cell mediators, reducing eosinophil activity, and decreasing cytokine release and neutrophil infiltration [77,78]. EP1 and EP3, in contrast, are pro-inflammatory, promote cough [79] and smooth muscle contraction, counteracting EP2 and EP4′s bronchodilation effects [77,78]. EP2 and EP4 receptor signaling promotes the accumulation of cAMP, whereas EP1 and EP3 receptors induce an increase in intracellular calcium [77,78].

Multiple clinical, experimental animal and cell models have demonstrated that PGE_2_ exerts significant antiallergic and anti-inflammatory effects [80,81,82]. PGE_2_ acts as a critical homeostatic “brake” on allergic responses, preventing the release of inflammatory mediators such as cysteinyl leukotrienes from mast cells [80,81,82]. This occurs primarily through the EP2 and EP4 receptors [80,81,82]. By diminishing Th2 polarization, PGE_2_ reduces the production of allergic cytokines like IL-5 and IL-13 and attenuates the recruitment of eosinophils to the site of an allergic reaction thereby reducing tissue inflammation [80,81]. In the airways, inhaled PGE_2_ induces smooth muscle relaxation and reduces bronchial hyperresponsiveness to allergens [83] and to exercise in asthma patients [84], and to aspirin in N-ERD [85]. Recent studies suggest that PGE_2_ deficiency predisposes individuals to more severe anaphylactic reactions [86].

In patients with CF, NPs exhibit a distinct inflammatory profile characterized by the upregulation of both COX-1 and COX-2 mRNA enzymes compared with healthy nasal mucosa and non-CF nasal polyps [87]. COX-2 protein also is strongly expressed in CF nasal polyps but is nearly undetectable in non-CF polyps or healthy nasal tissue [87]. This increased expression of both COX enzymes drives the high production of PGE_2_ typically seen in saliva [88], sputum [88], exhaled breath condensate [89], and urine [90,91,92] in CF patients.

Urinary concentrations of the main PGE_2_ metabolite, tetranor-PGEM (PGE-M), are significantly associated with more severe CFTR genotypes and severe clinical phenotypes, including higher pancreatic insufficiency and the presence of bronchiectasis and air trapping as shown on chest CT scans [91,92]. Notably, PGE_2_ levels in bronchoalveolar lavage fluid correlate positively with faster progression of lung disease, the higher the PGE_2_ levels, the faster the progression of the disease [93].

The deficient CFTR is directly associated with increased PGE_2_ synthesis as supported by the observation that treatment with CFTR modulators, such as ivacaft significantly reduced urinary PGE-M levels in patients with specific mutations (e.g., G551D) [94].

Research supports the concept that the enhanced COX expression and increased PGE_2_ production found in CF are directly related to CFTR dysfunction rather than to the presence of an inflammatory process associated with chronic bacterial infection [95,96]. Borot et al. [95] showed that CFTR inhibition leads to increased PGE_2_ release. Chen et al. [96] demonstrated that CFTR and PGE_2_ represent a critical feedback loop in epithelial biology, primarily influencing fluid secretion. In healthy cells, CFTR regulates PGE_2_, while PGE_2_ acts as a potent activator of CFTR. PGE_2_ binds to EP2 and EP4 and triggers an increase in intracellular cAMP, which activates Protein Kinase A (PKA), leading to the opening of the CFTR channel.

Interestingly, in airway epithelia, PGE_2_ can also stimulate chloride secretion through a CFTR-independent mechanism, mediated by Calcium-Activated Chloride Channels (CaCCs) driven by the EP4 receptor. Although typically associated with cAMP, EP4 activation can also trigger an increase in intracellular calcium which depends on extracellular calcium entry across the basolateral membrane with the contribution of Phosphoinositide 3-kinase, Src kinase, Serum glucocorticoid kinase 1 and ERKβ-catenin pathways. These findings suggest that PGE_2_ can use mechanisms to bypass airway chloride secretory defects in diseases associated with compromised CFTR function [97].

Collectively, these findings underscore the significant role of PGE_2_ in controlling the composition of ASL. In CF, there is a remarkable upregulation of PGE_2_ synthesis to levels not observed in other pathological conditions [88,89,90,91,92,93]. This extraordinary increase highlights the critical, yet ultimately unsuccessful, compensatory function of PGE_2_ in attempting to activate the impaired CFTR.

In marked contrast to CF, the regulation of COX pathways in non-CF nasal polyps presents an opposing pattern. Although it is generally assumed that the concentration of COX-1 remains stable under normal conditions, two to threefold increases in expression can occur in cells exposed to cytokines such as interleukin-1 beta (IL-1β), which is the primary cytokine involved in the upregulation of COX pathway in inflammatory processes [98]. Notably, IL-1β-stimulated NP fibroblasts from patients with N-ERD and non-N-ERD did not respond with the expected mild/moderate increase in COX-1 expression, which instead occurred in control nasal mucosa [99,100]. The lack of response of fibroblasts to an inflammatory stimulus suggests that COX-1 regulation is disturbed in the structural cells of NPs.

More striking is the difference between the marked upregulation of COX-2 in CF polyps compared to the significant downregulation of COX-2 expression observed in NP samples from non-CF NPs [99,100,101], particularly in those from N-ERD subjects [99,102,103,104]. The low COX-2 expression has also been demonstrated in IL-1β-stimulated NP fibroblast of N-ERD and non-NERD subjects [99,103,104] and airway epithelial cells from N-ERD [105].

This decrease in COX-2 expression is directly linked to a significant reduction in the synthesis of PGE_2_ in NPs compared with healthy nasal mucosa [99,101,103,104,106,107,108,109]. The reduction in PGE_2_ production is even more pronounced among individuals with N-ERD both in nasal tissue or in fibroblast and epithelial cells [99,101,106,107,109]. This diminished synthesis of PGE_2_ in non-CF nasal polyps is in stark contrast to the elevated PGE_2_ levels found in CF [88,89,90,91,92,93], highlighting a fundamental difference in the regulation of the COX pathway between these distinct diseases associated with CRSwNP (Table 1).

The mechanism behind COX-2 downregulation and reduced PGE_2_ production in NPs is not fully understood. Some studies indicate that IL-4 (T2 cytokine) and IFNγ (T1 cytokine) can lower COX-2 expression in healthy nasal mucosa mimicking the COX-2 pathway changes found in NPs [110,111]. These findings suggest that different types of inflammation may be involved in COX-2 pathway alterations in NPs.

Corticosteroids have been shown to downregulate COX-2 expression in epithelial nasal cells in vitro studies [112]. Conversely, in vivo research indicates that corticosteroids elevate COX-2 expression levels in NPs, further emphasizing the significant role of nasal inflammation in modulating COX-2 down-regulation [113].

With some discrepancies among studies in part depending on the methodology used (immunochemistry versus RT-PCR or western blot) and cells studied [114], downregulation of EP2 has been identified in NPs from N-ERD subjects [104,114,115,116,117]. This impaired receptor function further diminishes the already reduced capacity of PGE_2_ production to regulate CFTR potentially contributing to the development and progression of more severe NP growth.

In contrast to the relationship observed in CF patients, where elevated levels of PGE_2_ are associated with increased clinical severity [91,92,93], a different pattern emerges in individuals with non-CF NPs. Among these patients, lower concentrations of PGE_2_ are linked to a higher frequency of NP recurrence and an increased need for surgical interventions [118]. This trend is particularly pronounced in patients with N-ERD [119], who consistently demonstrate the lowest levels of PGE_2_ among NP subgroups [99,101,106,107,109]. These findings further highlight a fundamental difference in the PGE_2_ synthesis between CF and non-CF NPs conditions (Table 1).

## 4. Discussion

According to Bernstein et al. hypothesis [19,20], NPs develop because of inflammation—stemming from infection or allergy—that damages the mucosal lining and disrupts ion channels. This process leads to the production of thick mucus, impaired mucociliary function, tissue edema, and ultimately the formation of polyps.

Before Bernstein’s research, Al-Bazzaz et al. [120], found that endogenous prostaglandins locally regulate ion transport and help maintain lung fluid balance. They proposed that impaired ion transport may be a common factor in many respiratory diseases.

Bernstein et al. [20] acknowledged Al-Bazzaz’s work in general terms, noting that unusual levels of inflammatory mediators could affect sodium and chloride channels in the nasal epithelium and polyps, but did not discuss prostaglandins’ specific role described by Al-Bazzaz.

Recent advances in ion exchange regulation on epithelial surfaces allow for a modern update to Bernstein’s hypothesis. Six key findings may contribute to clarify NP development: (1) CFTR’s and other ion channels role is better understood; (2) ionocytes, which express CFTR, have been identified; (3) inflammation has been linked to ionocyte traits and CFTR activity; (4) PGE_2_ is recognized as an important regulator of ion transport; (5) therapies now exist to enhance CFTR function; and (6) new biologics target proinflammatory cytokines involved in NP pathology.

Evidence linking *CFTR* gene alterations to nasal polyps [11,31,32,33,34], together with the regression of polyps after restoration of abnormal CFTR protein function [41,42,43,44,45,46,47], provides the conceptual starting point for the I^3^PGE_2_ hypothesis. This hypothesis proposes that non-CF NPs arise from the convergence of CFTR and other ion channel dysfunction, persistent inflammation, altered ionocyte function, and reduced PGE_2_ production.

**Figure 2:**
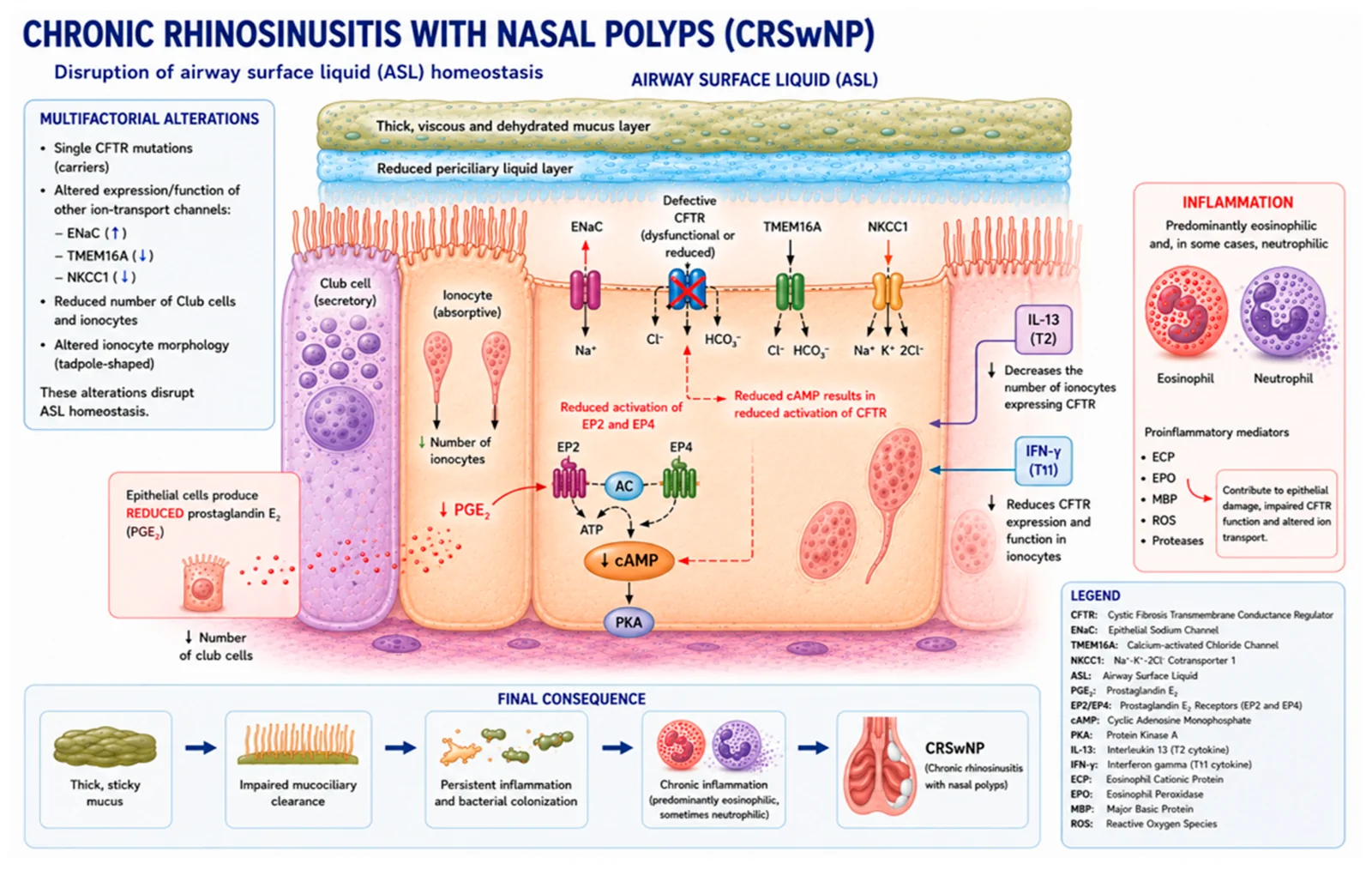
The I^3^PGE_2_ hypothesis suggests that the development of non-CF nasal polyps can be explained by an acquired dysfunction of CFTR and other ion channels, triggered by nasal epithelial injury caused by infections and irritants, which leads to chronic inflammation. In some patients, subtle CFTR gene alterations (heterozygous carrier status), together with inflammation-induced abnormalities in other ion channels associated with both type 1 and type 2 inflammation—including ENaC, TMEM16A, the Na^+^/K^+^/2Cl^−^ cotransporter 1 (NKCC1), and calcium-activated chloride channels (CaCCs)—may contribute to this process. Inflammation also reduces the number of CFTR-expressing ionocytes and alters their apical morphology. In addition, the damaged epithelium releases inflammatory cytokines such as IL-4 and IFN-γ, which downregulate COX-2 and markedly reduce PGE_2_ production, a key molecular signal for CFTR activation, thereby disrupting airway fluid balance. These changes promote a cycle of chronic inflammation, alterations in the local airway microbiome, thick mucus production, impaired mucociliary function, tissue edema, and ultimately polyp formation.

Minor *CFTR* gene alterations, including heterozygous carrier status [32,33,34], may make some patients more susceptible to additional insults such as inflammation. Dysregulation of other ion channels, including ENaC [49,50,51], TMEM16A [54,55], and NKCC1 [56], may further disturb ASL homeostasis in non-CF NPs. Inflammation may also decrease ionocyte number and impair ionocyte function, thereby compromising ion transport [75,76]. In non-CF polyps, particularly in patients with N-ERD, PGE_2_ production is markedly reduced [99,101,106,107,109] and EP2 receptor expression is downregulated [104,114,115,116,117]. Because the PGE_2_-EP2 pathway functions as a molecular key for CFTR activation, its impairment may result in functional CFTR silencing. Thus, the combined effects of ion channel dysfunction, altered ionocyte number and function, inflammation, and reduced PGE_2_ production may disrupt ASL balance in a way that parallels the dysfunction caused by CFTR mutations associated with NP growth (Figure 2). 

If the hypothesis is correct, to be effective NPs therapies should normalize ion channels activity by reducing NPs inflammation, reestablishing ionocyte density and function, and helping cells—especially epithelial cells and fibroblasts—normalize PGE_2_ production.

Systemic and topical intranasal corticosteroids (CS) are widely recognized for their effectiveness in treating NPs, with their main therapeutic benefit attributed to the suppression of eosinophilic inflammation [121,122]. Elevated levels of IL-13 are frequently observed within NPs [123]. CS inhibits IL-13 production in NPs [123], which could aid in restoring both ionocyte function and CFTR expression. However, the direct impact of CS on ionocytes remains uncertain and remains to be studied.

CS therapy raises COX-2 levels [113], which are low in NPs [99,100,101,103,104], Since COX-2 promotes PGE_2_ production and PGE_2_ activates CFTR [95,96], CS may restore ion balance by normalizing CFTR activity.

These mechanisms indicate that CS may benefit CRSwNP not only by reducing inflammation but also by restoring ion transport in the nasal epithelium, thereby supporting the management of NPs.

Biologics have transformed CRwNP treatment. Biologic therapies represent a more targeted approach compared to the broad immunosuppressive effects of CS.

Dupilumab, is a targeted biologic that reduces Type 2 inflammation by binding to the interleukin-4 receptor alpha (IL-4Rα) subunit. By blocking this shared receptor, it simultaneously blocks IL-4/IL-13 signaling, reduced eosinophlilic inflammation, quickly improves nasal congestion and smell, sometimes within three days [124,125]. It reduces the need for sinus surgeries and CS treatment [125]. Its effectiveness might stem from restoring ionocyte function by blocking IL-13′s harmful effects. Additionally, blocking IL-4Rα also helps normalize nasal fluid PGE_2_ levels [126], and thereby may restore aspirin tolerance in N-ERD patients [127]. Higher PGE_2_ is linked to better sense of smell [126]. A sub analysis showed that the N-ERD subgroup responded better to dupilumab than the non-N-ERD cohort [128]. Notably, dupilumab has proven to be effective in improving associated symptoms and reducing the size of NPs in patients with CF who have type 2 inflammation-related comorbidities such as atopy, further supporting the negative effects that inflammation exerts on ion channels normal activity [129,130].

Omalizumab binds to the domain of circulating free IgE, preventing it from attaching to high-affinity receptors on cells such as mast cells and basophils [131]. Although it does not directly act on IL-13 or IL-4, omalizumab can indirectly decrease its release [132]. In vitro research demonstrated that omalizumab lowers cysteinyl leukotriene production by dissociating IgE already bound to mast cells and basophils [131]. Clinical trials show omalizumab reduces polyp size, improves nasal symptoms and sense of smell, decreases the need for corticosteroid rescue therapy, and lowers the risk of nasal polyp surgery [133,134].

Omalizumab is associated with clinical improvements in patients with N-ERD [135,136]. Importantly, omalizumab restores partial or total aspirin tolerance associated with rapid decline of PGD-M, the urine metabolite of PGD_2_ (a proinflammatory and bronchoconstrictor prostaglandin) and leukotriene E4 (LTE4) (terminal cysteinyl leukotriene metabolite), both mainly derived from mast cells [135,136]. However, PGE_2_ levels were not reported, so it remains uncertain if changes in PGD_2_ and LTE4 are due to increased PGE_2_ production. It is still unclear whether omalizumab’s effectiveness in treating NPs involves significant effects on IL-4 and IL-13 levels, as well as on ionocyte function.

TSLP is an alarmin belonging to the IL-2 family, which is released in response to environmental triggers like allergens, microbes, and pollutants. TSLP activates dendritic cells and promotes T2 cell differentiation, leading to recruitment of eosinophils and other inflammatory mediators. Increased TSLP expression in NPs correlates with higher eosinophil counts and type 2 cytokines (IL-4, IL-5, IL-13) [137,138]. Tezepelumab, a biologic targeting TSLP receptor, shows efficacy in both T2 and some non-T2 inflammation patients, previously lacking targeted treatments [139].

Tezepelumab therapy in CRSwNP is very effective in shrinking polyps and improving congestion and smell scores, while reducing the need for surgery and systemic CS. Its benefits were consistent regardless of blood eosinophil levels or comorbid conditions such as asthma or N-ERD [140,141,142]. Unlike dupilumab, which directly blocks the receptors for IL-4 and IL-13, tezepelumab stops the signal that activates cells such as group 2 innate lymphoid cells (ILC2s) to produce IL-4 and IL-13 [139]. Current scientific literature does not indicate that tezepelumab directly affects the production of PGE_2_. Instead, research highlights a reverse relationship where PGE_2_ acts as a downregulator of TSLP [143]. Although there is no evidence that tezepelumab directly affects ionocytes or CFTR, it may indirectly support COX-2 expression and ionocyte function by inhibiting cytokine IL-4 and IFN-γ. Since IL-4 and IFN-γ can reduce COX-2 expression [111,112] and IL-13 can decrease CFTR-expressing ionocytes [73], these mechanisms might partly explain Tezepelumab’s efficacy.

Mepolizumab is effective in treating NPs primarily by selectively targeting and neutralizing IL-5, a key cytokine in the type 2 inflammatory pathway. According to results from clinical trials, this biological mechanism translates into significant clinical benefits: Reduction in polyp size, improvement in nasal obstruction, congestion, and loss of sense of smell, decreased requirement for rescue systemic CS and revision nasal surgeries [144,145]. In contrast with dupilumab, mepolizumab was not associated with increase in nasal PGE_2_ and urinary PGEM in N-ERD patients [146].

Depemokimab is the first ultra-long-acting anti-IL-5 biologic that allows biannual dosing. In trials, it reduced nasal polyp scores and obstruction more than placebo, but its clinical benefits are limited compared to other biologics [147].

Unlike mepolizumab, which targets and neutralizes the IL-5 protein, benralizumab attaches to the IL-5 receptor on eosinophils, resulting in a swift and almost complete removal of these cells from both the bloodstream and NP tissue. In a clinical trial, benralizumab led to significant reductions in polyp size and blockage when compared to placebo. However, compared to other major biologics, it usually provides the least improvement in nasal congestion and sense of smell [148].

Clinical comparisons consistently indicate that dupilumab is generally the most effective biologic for nasal polyps, often followed by tezepelumab and omalizumab with mepolizumab and benralizumab ranking lower [149,150,151,152,153,154].

The I^2^PGE_2_ hypothesis should also encompass non-CF subtypes of NPs, including those occurring in patients with host defense deficiencies such as primary antibody deficiencies and primary ciliary dyskinesia (PDC) [155,156,157]. In these populations, chronic infection and inflammation may alter cytokine profiles towards a non-eosinophilic pattern (e.g., IFN-γ), which is associated with reduced ionocyte numbers and diminished CFTR expression, thereby contributing to the development of NPs [75]. Nasal mucosa from PCD patients were characterized by impaired basal chloride secretion and chloride secretory responses to forskolin, suggesting that according to the hypothesis abnormal regulation of ion channels is a common finding in all types of NPs [158].

## 5. Conclusions

Genetic disorders such as CF validate the crucial role that ASL homeostasis plays in individuals that suffer from CRSwNP. Based on this observation, the I^3^PGE_2_ hypothesis suggests that the ionic composition of nasal secretions and the underlying transport mechanisms are significantly altered in patients with non-CF nasal polyps. These changes mimic the airway fluid homeostasis failure seen in severe CFTR mutations and are the primary driver of the swelling that characterizes polyp formation.

This hypothesis has several important limitations. First, direct evidence linking dysfunction of ion channels such as ENaC, TMEM16A, and NKCC1 to CRSwNP development remains very limited. Second, human data connecting ionocyte dysfunction with CRSwNP progression are still scarce. Third, the differential effects of T2 and non-T2 inflammation on ion channel and ionocyte function remain poorly defined. Fourth, it is not known whether treatments such as corticosteroids and biologics can restore ion channels and ionocyte function. Finally, longitudinal mechanistic studies are still lacking.

In summary the proposed pathway—from ion channel abnormalities, inflammation, ionocyte dysfunction, and reduced PGE_2_ production to nasal polyp development—integrates established observations with assumptions that still require further validation.

The studies needed to validate the hypothesis include: (1) In-depth study of the role played by the different ion channels involved in the control of ASL. (2) Evaluation of the role of different types of inflammation in the regulation of ion channel function. (3) Clarification of the role of ionocytes, which are notable for being the major carriers of CFTR. (4) Evaluation of the efficacy of corticosteroids and biological therapies in the treatment of CRSwNP and CRSsNP by: (a) their anti-inflammatory effects, particularly those regulated by the cytokines IL-4 and IL-13; (b) their ability to restore ionocyte function; and (c) normalize PGE_2_ synthesis through increased COX-2 activity.

These studies would benefit from a combined methodological approach, including: (1) single-cell RNA sequencing and lineage tracing to characterize airway cell composition in CRSwNP before and after treatments [61,62]; and (2) specialized methods used to assess ion channel’s function, such as electrophysiological and ex vivo bioelectric techniques, nasal potential difference measurements, and primary nasal epithelial cells grown as organoids [159].

If confirmed, this hypothesis would explain how a shared pathogenic pathway can produce clinically distinct CRSwNP phenotypes, including infectious, allergic, eosinophilic, non-eosinophilic, fungal, immunodeficiency-associated, and mucociliary dysfunction-related forms. It may also help account for differences in treatment response. By identifying the dominant mechanism(s) driving CRSwNP in each patient, this framework could guide preventive strategies and more personalized therapies.

## Figures and Tables

**Table 1:** Differences between Cystic Fibrosis and Non-Cystic Fibrosis CRSwNP.

Parameter	Cystic Fibrosis	Non-Cystic Fibrosis	Non-CF/N-ERD
**PGE_2_ synthesis**	Marked increase	Mild to moderate decrease	Marked decrease
**Ion channels**			
CFTR	Moderate to severe alteration	Occasional mild to moderate alterations *	Not reported
ENaC	Not reported	Increased expression	Not reported
TMEM16A	Not reported	Reduced expression	Not reported
NKCC1	Not reported	Increased expression	Not reported
**Ionocytes**			
Number	No change	No change	Not reported
CFTR	Reduced expression	Reduced expression	Not reported
Morphology	No change	Loss of tadpole-like morphology	Not reported

* CFTR-related disorders, congenital bilateral absence of the vas deferens (CBAVD), and single CFTR mutations (carriers). ENaC, epithelial sodium channel; TMEM16A, transmembrane member 16A (also known as anoctamin-1); NKCC1, Na^+^/K^+^/2Cl^−^ cotransporter 1; CRSwNP, chronic rhinosinusitis with nasal polyps; N-ERD, NSAID-exacerbated respiratory disease.

## Data Availability

No new data were created or analyzed in this study. Data sharing is not applicable to this article.

## Abbreviations

ASL	Airway surface liquid
CaCCs	Calcium-Activated Chloride Channels
CCAD	Central compartment atopic disease
CF	Cystic fibrosis
CFTR	Cystic Fibrosis Transmembrane Conductance Regulator
COX	Cyclooxygenase
CRS	Chronic rhinosinusitis
CS	Corticosteroid
ENaC	Epithelial Sodium Channel
IL	Interleukin
ILC2s	Type 2 innate lymphoid cells
N-ERD	Non-steroidal anti-inflammatory drug
NKCC1	Na^+^/K^+^/2Cl^−^ cotransporter 1
NP	Nasal polyp
PDC	Primary ciliary dyskinesia
PG	Prostaglandin
SPLUNC1	Secreted protein short palate lung and nasal epithelial clone 1 SPLUNC1
TSLP	Thymic stromal lymphopoietin
